# Priorities in updating training paradigms in orthopedic manual therapy: an international Delphi study

**DOI:** 10.3352/jeehp.2023.20.4

**Published:** 2023-01-27

**Authors:** Damian Keter, David Griswold, Kenneth Learman, Chad Cook

**Affiliations:** 1 Department of Veterans Affairs Medical Center, Cleveland, OH, USA; 2 Department of Graduate Studies in Health and Rehabilitation Sciences, Youngstown State University, Youngstown, OH, USA; 3 Department of Orthopaedic Surgery, Duke University School of Medicine, Durham, NC, USA; 4 Department of Population Health Sciences, Duke University, Durham NC, USA; 5 Duke Clinical Research Institution, Duke University, Durham, NC, USA; Hallym University, Korea

**Keywords:** Musculoskeletal manipulations, Health education, Spinal manipulation

## Abstract

**Purpose:**

Orthopedic manual therapy (OMT) education demonstrates significant variability between philosophies and while literature has offered a more comprehensive understanding of the contextual, patient specific, and technique factors which interact to influence outcome, most OMT training paradigms continue to emphasize the mechanical basis for OMT application. The purpose of this study was to establish consensus on modifications & adaptions to training paradigms which need to occur within OMT education to align with current evidence.

**Methods:**

A 3-round Delphi survey instrument designed to identify foundational knowledge to include and omit from OMT education was completed by 28 educators working within high level manual therapy education programs internationally. Round 1 consisted of open-ended questions to identify content in each area. Round 2 and Round 3 allowed participants to rank the themes identified in Round 1.

**Results:**

Consensus was reached on 25 content areas to include within OMT education, 1 content area to omit from OMT education, and 34 knowledge components which should be present in those providing OMT. Support was seen for education promoting understanding the complex psychological, neurophysiological, and biomechanical systems as they relate to both evaluation and treatment effect. While some concepts were more consistently supported there was significant variability in responses which is largely expected to be related to previous training.

**Conclusion:**

The results of this study indicate manual therapy educators understanding of evidence-based practice as support for all 3 tiers of evidence were represented. The results of this study should guide OMT training program development and modification.

## Introduction

### Background

Clinical research suggests orthopedic manual therapy (OMT) provides comparable or superior effects for reducing pain in individuals with musculoskeletal disorders [1]. Mechanisms research outlines similar effects with all forms of manual therapy techniques [2]. OMT techniques vary per post-graduate training philosophy; however, frequently the operator targets specific joints, respects biomechanical concepts, and targets the force-based manipulation to the region of dysfunction [3]. Given the focus on specificity, the training time required to gain “mastery” can be significant. Interestingly, a review investigating the specificity of joint mobilization shows a specific versus randomly applied technique provides similar outcomes [4]. Another review demonstrated similar findings with joint manipulation [5]. Moreover, data suggest that a remotely applied manipulation may be more beneficial than a locally applied technique [6]. While technique specificity is proving to be less crucial, literature supports the importance of contextual factors and patient factors on OMT outcomes [7]. Studies have suggested that contextual factors (e.g., patient characteristics, practitioner characteristics, treatment characteristics, therapeutic alliance, and clinical setting) may be more important in determining treatment outcomes than the technique [5]. This new found information highlights the need to investigate whether a modification of a traditional advanced training paradigm for OMT is needed. Whereas customary training strategies such as (1) handling competency and (2) understanding risk of harm will never be outdated, additional training elements such as (3) communication of what to expect with the technique, and (4) recognition of when a technique appears to be beneficial versus not, are skills that deserve consideration.

### Objectives

This study aims to establish consensus on modifications to training paradigms within post-graduate OMT education through Delphi study. Once consensus methods are identified, the potential of implementing these methods into training programs may increase.

## Methods

### Ethics statement

Institutional Review Board approval was obtained through Youngstown State Universities Institutional Review Board (2022-204) prior to data collection. Informed consent was obtained electronically via participants clicking URL to the questionnaire.

### Setting

This Delphi was completed electronically from July 2022–November 2022.

### Study design

A 3-round Delphi study following recommended guidelines for conducting and reporting of Delphi studies (CREDES) was performed [8].

### Respondent group

A priori goal of 30 participants completing all 3 rounds of the instrument was set as this has been recommended to be representative and feasible in qualitative Delphis [9]. A panel of experts included international participants with advanced manual therapy education demonstrated through either completion of an International Federation of Orthopaedic Manipulative Physical Therapists (IFOMPT) recognized fellowship in OMT or completion of an academic doctorate with research specialization related to OMT. Educators were identified through web search of both IFOMPT and associated national fellowship databases. Individuals were sought whom teach advanced manual therapy within fellowship, residency, or other advanced post-doctoral training programs.

### Workgroup

Four individuals, including the primary investigator and 3 individuals experienced in qualitative research. All workgroup members were physical therapists with 9 to 33 years of clinical experience. Three workgroup members were mixed-methods researchers with experience in the Delphi method.

### Instrumentation: 3-round web-based Delphi using Qualtrics survey system

Round 1 was an open-ended design developed to identify opinions/perceptions on the future of manual therapy training paradigms. Round 1 identified basic demographics including experiences experts had with training programs. Open-ended questions asking participants to identify recommended training paradigms for manual therapy techniques was implored. Face validity was investigated through a pilot survey of 5 individuals with qualifications to participate in the study whom were not included in final data collection [8].

Following Round 1, the workgroup examined each individual response and utilized qualitative thematic coding (Supplement 1). Round 2 included a list of the themes derived from Round 1 questioning. Respondents utilized a 4-point Likert scale (strongly agree, agree, disagree, and strongly disagree) to score each of these themes by level of agreement with the recommended training paradigm.

Round 3 included the same themes and grading scales as Round 2 with the addition of graphs representing the descriptive statistical scores computed from Round 2. With this information available, the respondents were asked to rescore each item on the same 4-point Likert scale. All responses were de-identified before data analysis by removing columns containing identifiable data from report.

### Protocol

**Fig. 1.**
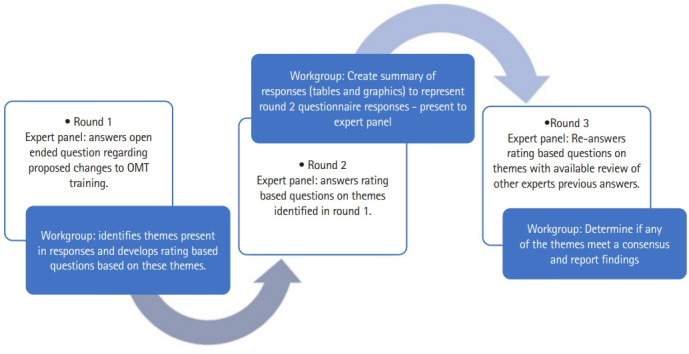
Flow chart for current study protocol (3-round Delphi) including expert panel and workgroup duties. OMT, orthopedic manual therapy.

Protocol information is provided in Supplement 1. Study protocol and summarized in Fig. 1.

### Data analysis

**Fig. 2.**
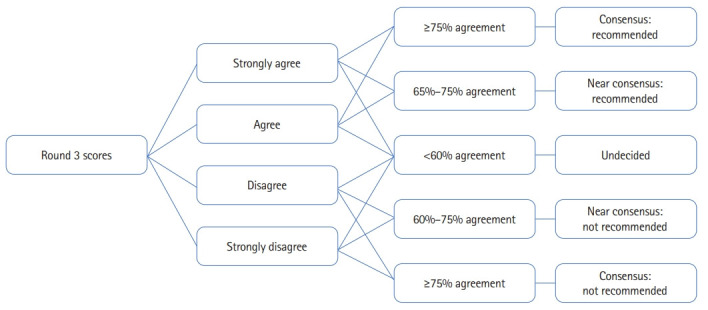
Flow chart indicating how levels of consensus were obtained following round 3.

IBM SPSS ver. 29.0 (IBM Corp.) was used for all quantitative analyses. Scores for Round 3 were divided into 2 categories based on descriptive identifiers: The tally of ‘‘strongly disagree’’ and ‘‘disagree’’ represent the percentage of scores in the “not recommended” category, meaning that the proposed training paradigm is not recommended. On the contrary, the tally of “strongly agree” and “agree” represented the percentage of scores in the “recommended” category, meaning that the proposed training paradigm is recommended. Consensus was determined a priori if 75% or greater of the respondents score the component of education as either “not recommended” or “recommended” [8]. When an item did not reach consensus, the decision was made between “near-consensus” and “undecided”. Agreement between 60%–75% either for “recommended” or “not recommended” was considered “near consensus” while agreement less than 60% was considered “undecided”. The process of determining consensus status is further outlined in Fig. 2. A composite score for each component of training was calculated based on the following formula:

[n1×(-2)]+[n2×(-1)]+[n3×1]+[n4×2]

n1: number of respondents answering “strongly disagree” with component of training

n2: number of respondents answering “disagree” with component of training

n3: number of respondents answering “agree” with the component of training

n4: number of respondents answering “strongly agree” with the component of training

Sum of individual composite scores was used to establish a combined composite score. The higher the combined composite score, the more important the training paradigm to manual therapy education. Mann-Whitney U statistics assessed differences in scores between Round 2 and Round 3.

## Results

One-hundred sixty-four educators were identified and invited to participate representing 4 countries (United States, Canada, United Kingdom [England], New Zealand). Advanced degrees included Doctor of Science (DSc), Doctor of Philosophy (PhD), and Fellowship training (American Academy of Orthopedic Manual Physical Therapy [FAAOMPT]; Musculoskeletal Association of Chartered Physiotherapists [FMACP]; New Zealand Manipulative Physiotherapists Association [FNZMPA]; Canadian Academy of Manipulative Physiotherapy [FCAMPT]).

Forty-one participants responded to Round 1 for a response rate of 25% (Table 1, Dataset 1). Thematic coding of responses produced 25 themes for OMT training foci (Table 2); 19 themes for what should be omitted from focus within OMT education (Table 3); and 37 themes for foundational knowledge needed to apply OMT (Table 4). The themes were agreed upon by all workgroup members. Thirty-three individuals completed Round 2 (80.5% retention rate) for a 20.1% overall response rate (Dataset 2). Results of Round 2 were presented to the same 33 respondents to re-score the same themes with 28 of the 33 respondents completing the third and final round (Dataset 3). Retention rate between Round 2 and Round 3 was 84.8% and 17.1% overall response rate.

Question 1 investigated which areas should be focused on within OMT education with consensus reached supporting all 25 themes (Table 2). Composite scores representing the strength of the recommendations are provided with factors including patient comfort, patient handling, safety, and ability to modify techniques as needed having some of the strongest recommendations. Other patient specific factors including communication and managing patient expectations were also ranked highly. Technique specific factors including ability to grade mobilization, localization of tissue dysfunction, use of OMT for soft tissue dysfunction, and motor control did not have the same strength of recommendation although they did reach consensus. Education on both neurophysiological and psychological mechanisms associated with OMT scored higher than education on the biomechanical mechanisms associated with OMT. Use of OMT as part of multimodal care plan also rated highly amongst the panel of experts. Limited variance was seen between respondents (mean standard deviation [SD]=0.60, mean variance=0.38).

Question 2 investigated what areas of focus should be omitted from OMT education with only one of the themes (visceral manipulation) reaching consensus (Table 3). Several other themes including complex reasoning, non-reliable assessment techniques, terminology attempting to differentiate philosophies, rigidly defined non-adaptive techniques, non-evidence-based treatments, and treatment based purely off a research driven model produced near consensus results supporting omitting (60%–75% agreement). Themes including pain neuroscience education, segment localization, and treatment direction based on arthrokinematics produced near consensus results against omitting (60%–75% agreement). Moderate variance was seen between respondents for this question (mean SD=0.94, mean variance=0.90).

Question 3 investigated what foundational knowledge is needed to apply OMT. Thirty-four of the 37 themes met consensus supporting (Table 4). Three themes were near consensus supporting including use of grading scales, histology, and understanding the SINSS model (Severity, Irritability, Nature, Stage, Stability), and 1 theme was undecided (ability to lock out joints). Strongest recommendations were towards patient safety, indications and contraindications, patient-centered care, strong communication skills, patient education as an adjunct to OMT, strong assessment and evaluation skills, ability to obtain a good history, ability to adapt techniques to specific patients, utilization of patient response model, following OMT with functional movement and exercise, and understanding of anatomy. Minimal variance was seen between respondents for this question (mean SD=0.53, mean variance=0.30).

No significant difference was found between Round 2 and Round 3 composite scores for Question 2 assessing themes to omit from OMT education (P=0.872, U=175). Question 1 (P=0.013, U=185) and question 3 (P=0.002, U=403) both showed significant differences between Round 2 and Round 3 composite scores.

## Discussion

### Interpretation

The Delphi method is a recommended tool for achieving consensus in medical education [10]. The validity of the consensus achieved within Delphi studies largely rests on the quality of the experts, which develop the consensus. The participants demonstrated advanced manual therapy knowledge through appropriate higher-level credentials, and who were involved in training within manual therapy programs.

### Manual therapy training should focus on

All 25 themes from Round 1 reached consensus to be included within OMT education. Patient factors all rated highly amongst the participants. This aligns with published clinical trials that have shown the moderating effect of comfort, therapeutic alliance, and expectations on OMT outcomes [11]. A lesser focus on the biomechanical mechanisms was observed with higher scoring for focus on both the neurophysiological and psychological mechanisms. Previous models have outlined these mechanisms as they relate to OMT outcomes [12]. Utilizing OMT as part of a multimodal care plan ranked highly aligning with a recent high-level review finding this to be a consistent recommendation across practice guidelines [13]. The importance of advanced assessment skills and the ability to identify responders and non-responders ranked highly; however, localization of tissue dysfunction had significantly less strength of a recommendation. Themes including biomechanics, arthrokinematics, osteokinematics, neuromuscular training, and pain science all ranked moderately with similar composite scores. The overall high consensus rate of presented themes supports the perceived importance of incorporating education on a vast array of topics within OMT educational paradigms.

### Manual therapy training should omit focus on

Nineteen themes were identified in Round 1; however, only 1 of those themes met consensus to omit from OMT education. The contradiction among respondents’ answers likely corresponds to differing OMT philosophies. Some were strongly opposed to omitting biomechanical principles (biomechanical effects of OMT [17.9%]; arthrokinematics & osteokinematics [17.9%]; treatment based on biomechanical findings [21.4%]; treatment direction based on arthrokinematics [14.3%]) while others were strongly in favor of omitting these same principles (biomechanical effects of OMT [21.4%]; arthrokinematics & osteokinematics [14.3%]; treatment based on biomechanical findings [17.9%]; treatment direction based on arthrokinematics [14.3%]).

Omitting focus on visceral manipulation education was the only theme to meet consensus with 78% agreement. A recent review suggests a lack of quality unbiased studies demonstrating efficacy in this domain [14]. Some support for omitting focus on treatment without evidence, complex reasoning, application of technique without clinical reasoning, and non-reliable assessment techniques; however, these did not reach a consensus. These results align with the overall response slightly (near-consensus) leaning towards omitting treatment based off a purely research-driven model and show agreement with previous reviews demonstrating limited compliance with research-based guidelines [15]. These findings were further supported by the 94.4% agreement for the evidence-based practice model (research+clinical expertise+patient preference and values) to be a focus within OMT education.

### The foundational knowledge necessary to apply manual therapy

Thirty-four of the 37 themes from Round 1 reached a consensus. One undecided theme emerged involving the ability of individuals to lock out joints. The rating of this theme was variable with opposing positions to this requirement. Themes supporting patient-centered care, communication, therapeutic alliance, and patient safety all ranked amongst the strongest recommendations. This aligns with responses to question 1 and with the aforementioned literature supporting the importance of the therapeutic alliance in OMT outcomes.

### Limitations

There are limitations related to a true representation of the sample population. With significant variation between manual therapy philosophies, some may not be represented within this sample and others may have differing opinions from this panel of experts. While we attempted to be representative by including international participants, a significant proportion was stationed within the US IFOMPT accredited education programs are represented across 25 countries; however, most of these programs did not report faculty members and contact information on the associated websites; therefore, several countries were not appropriately represented.

### Generalizability

Given the international representation of this study along with fair representation of different OMT philosophies these results can be relatively generalized to post-graduate manual therapy education including continuing education, advanced manual therapy certification and fellowship training; however, care should be taken given the above stated limitations related to geographical restrictions.

### Suggestions for future studies

Future studies should attempt to understand the reasoning behind the overwhelming consensus related to included themes and the minimal consensus on excluded themes. Furthermore, given the proposed changes in training paradigms future studies should identify if these same principles indicate a shift in the clinical application of OMT.

### Conclusion

The combined high consensus rate for themes to focus on within OMT education along with the low consensus rate of themes to omit focus on within OMT education stresses the breadth of knowledge which appears to be pertinent to OMT. Of interest was that while 91% of respondents supported focus on training related to biomechanical mechanisms, 60% supported omitting treatment based on biomechanical findings and 40% supported omitting training on segments localization. This suggests that the biomechanical effect should be more of a focus than the biomechanical rationale for applying the technique. The included themes were developed by the respondents; however, variability in interpretation of the themes, along with differences seen within OMT training paradigms likely contributes to this discrepancy. Future studies should look to differentiate which biomechanical findings are viewed as important versus not in OMT assessment.

Overall support was seen for education promoting understanding the complex psychological, neurophysiological, and biomechanical systems as they relate to evaluation and treatment effect. The support for care based on all aspects of evidence-based practice model supports patient centered care and the understanding of complex interactions surrounding manual therapy intervention.

## Figures and Tables

**Table 1.** Respondent demographics, years’ experience in research and clinical practice, education/training provided, education/training received

Characteristic	No. (%)
Age (yr)	
30–40	6 (14.6)
40–50	16 (29.0)
50–60	14 (34.1)
>60	5 (12.2)
Gender	
Male	31 (75.6)
Female	10 (24.4)
Years’ experience in research (yr)	
None	8 (19.5)
0–5	9 (22.0)
5–10	10 (24.4)
10–15	6 (14.6)
15–20	3 (7.3)
>20	5 (12.2)
Years’ experience in clinical practice (yr)	
None	0
0–5	0
5–10	2 (4.9)
10–15	6 (14.6)
15–20	8 (19.5)
>20	25 (61.0)
Education/training:	
Post-doctoral degree (DSc, PhD, etc.)	20 (36.4)
Fellow (AAOMPT, etc.)	35 (63.6)
Type of post-doctoral manual therapy training provided	
Residency (OCS, SCS, etc.)	17 (21.0)
Fellowship (FAAOMPT, etc.)	36 (44.4)
Continuing education	28 (34.6)
Philosophies trained under	
Patient response model (Maitland, Mckenzie, Mulligan)	10 (25.6)
Biomechanical/Arthrokinematic Model (Ola Grimsby, NAIOMPT, Paris, Kaltenborn, Osteopathic)	9 (23.1)
Mixed training	18 (46.2)
No response	2 (5.1)

DSc, Doctor of Science; PhD, Doctor of Philosophy; AAOMPT, American Academy of Orthopaedic Manual Physical Therapy; OCS, Orthopaedic Clinical Specialist; SCS, Sports Clinical Specialist; FAAOMPT, Fellow of American Academy of Orthopaedic Manual Physical Therapy; NAIOMPT, North American Institute of Orthopedic Manual Physical Therapy.

**Table 2.** Question 1: Round 2 and Round 3 composite scores, and consensus status

I would recommend that manual therapy training should focus on...	Round 2 composite scores	Round 3 composite scores	Round 3 consensus status
Patient self-reported outcomes and ability for clinicians to assess them	32	28	C-R
Neurophysiological mechanisms associated with OMT including the effect of touch	57	48	C-R
Psychological mechanisms associated with OMT	47	43	C-R
Biomechanical mechanisms associated with OMT	42	33	C-R
Patient-centered care (communication)	59	47	C-R
Patient-centered care (therapeutic alliance)	51	44	C-R
Pain neuroscience education	40	35	C-R
Managing patient expectations	43	48	C-R
Addressing lifestyle behaviors to promote overall wellness	39	40	C-R
Use of OMT as part of multimodal care plan	63	52	C-R
Application of EBP (patient preference, therapist preference/skill, research)	58	45	C-R
Use of OMT for soft tissue and fascial problems	31	26	C-R
Use of OMT for non-pain uses (motor control, tone reduction)	27	21	C-R
Determining candidates for MT (localization of tissue dysfunction)	34	26	C-R
Determining candidates for MT (identification of responders and non-responders)	55	41	C-R
Psychomotor skills	54	47	C-R
Patient handling	56	50	C-R
Advanced assessment skills	56	42	C-R
Patient comfort	56	50	C-R
Safety	59	51	C-R
Ability to modify techniques as needed	59	52	C-R
Ability to grade mobilizations	40	26	C-R
Biomechanics, osteokinematics, and arthokinematics	40	39	C-R
Neuromuscular training	50	37	C-R
Pain science	49	40	C-R

C-R, consensus-recommended; OMT, orthopedic manual therapy; EBP, evidence-based practice; MT, manual therapy.

**Table 3.** Question 2: Round 2 and Round 3 composite scores, and consensus status

I would recommend that manual therapy training should omit focus on...	Round 2 composite scores	Round 3 composite scores	Round 3 consensus status
Terminological and philosophical considerations of different OMT philosophies	0	7	UN
Biomechanical effects of OMT	-4	-1	UN
Complex reasoning that is not observable/reproduceable	2	17	NC-R
Clinical prediction rules	-1	9	UN
Visceral manipulation	30	24	C-R
Pain neuroscience education	-14	-8	NC-NR
Application of technique without clinical reasoning	3	12	UN
Resetting of nervous system with manipulation techniques	17	3	UN
OMT for treatment of non-pain/motion complaints	7	1	UN
Terminology attempting to differentiate philosophies (school of thought)	20	12	NC-R
Arthrokinematics/osteokinematics	-1	-1	UN
Non-reliable assessment techniques (palpation, sacroiliac joint innominate)	23	15	NC-R
Segment localization	-1	-3	NC-NR
Treatment based on biomechanical findings	-5	-5	UN
Treatment direction based on arthrokinematics	6	-6	NC-NR
Treatment based on clinical prediction rules	9	6	UN
Rigidly defined techniques that are not adaptive to patient needs	12	17	NC-R
Treatment “fads” without evidence supporting	28	21	NC-R
Treatment based purely off research driven model	-3	10	NC-R

OMT, orthopedic manual therapy; UN, undecided; NC-R, near consensus-recommended; C-R, consensus-recommended; NC-NR, near consensus-not recommended.

**Table 4.** Question 3: Round 2 and Round 3 composite scores, and consensus status

The foundational knowledge I feel is necessary to apply manual therapy is...	Round 2 composite scores	Round 3 composite scores	Round 3 consensus status
Anatomy	62	50	C-R
Neurophysiology	53	47	C-R
Arthrokinematics/osteokinematics	37	30	C-R
Relationship between physiology and neuromuscular system	51	40	C-R
Histology	10	11	NC-R
Epidemiology	20	24	C-R
History of OMT	19	16	C-R
Current state of OMT	30	28	C-R
Philosophies of OMT	20	18	C-R
Grading scales	22	15	NC-R
Understanding of SINSS model	30	18	NC-R
Mechanisms of OMT response	56	46	C-R
Manual therapy application based on pain mechanism (mechanism based OMT)	53	45	C-R
Understanding lack of specificity in OMT	45	39	C-R
Indications/contraindications	64	53	C-R
Patient safety	63	53	C-R
Patient education as adjunct to OMT	61	50	C-R
Following OMT with functional movement/exercise	60	52	C-R
Understanding exercise science	52	42	C-R
Eclectic skill set (fascial, soft tissue, neural, articular)	26	31	C-R
Ability to identify impairments and functional limitations	56	46	C-R
Ability to obtain good history	62	54	C-R
Patient-centered care	63	53	C-R
Patient response model (test-retest)	62	50	C-R
Strong assessment/evaluation skills	62	52	C-R
Strong communications skills	65	53	C-R
Pattern recognition	56	47	C-R
Understanding cognitive and psychological contributors to pain and stiffness	56	46	C-R
Exercise prescription	58	44	C-R
Application of the biopsychosocial model	51	44	C-R
Evidence-based practice	57	46	C-R
Identifying gaps within the literature	43	41	C-R
Ability to critique research methodology	48	40	C-R
Technique	50	47	C-R
Psychomotor skills	52	44	C-R
Ability to adapt techniques to specific patients	61	51	C-R
Ability to lock out joints	13	9	UN

C-R, consensus-recommended; NC-R, near consensus-recommended; OMT, orthopedic manual therapy; SINSS, Severity, Irritability, Nature, Stage, Stability; UN, undecided.

## Footnotes

**Data availability**
Data files are available from Harvard Dataverse: https://doi.org/10.7910/DVN/XAMMVU Dataset 1. Raw response data at the 1st round Delphi survey from 57 participants from which 16 responses were not included due to incomplete response.

Dataset 2. Raw response data at the 2nd round Delphi survey from 36 participants from which 3 responses were not included due to incomplete response.

Dataset 3. Raw response data at the 3rd round Delphi survey from 29 participants from which 1 response was not included due to incomplete response.

## Supplementary materials

Supplementary files are available from Harvard Dataverse: https://doi.org/10.7910/DVN/XAMMVU

Supplement 1. Study protocol.

Supplement 2. Audio recording of the abstract.
