# Supplementary material for: Priorities in updating training paradigms in orthopedic manual therapy: an international Delphi study
Source: J Educ Eval Health Prof. 2023 Jan 27;20:4. doi: 10.3352/jeehp.2023.20.4 (PMC9993014; doi:10.3352/jeehp.2023.20.4)
Supplement: Supplementary file 4 — Supplement 1. Study protocol. [file Jeehp-20-04-suppl1.docx]

**Supplementary material**

**Supplement 1. Study protocol**

Invitations for participation were distributed to the identified experts through email including information on the purpose of the study, how they were selected as expert panelists, and information on informed consent. They also received a web-based link to the online survey. Participants who did not respond to the initial request for participation were emailed a second time 14 days after the initial email as a reminder to encourage participation. Respondents consented to participate by following the provided web link to the Round 1 questionnaire through the Qualtrics web-based survey system. The questionnaire was stored on a password-protected server through Qualtrics software. This company is a common vendor used for survey research and has significant data protection policies in place.

The purpose of Round 1 was to allow participants to identify content that they consider to be most important to include or omit from post-graduate manual therapy educational models, along with identifying baseline knowledge that they feel necessary to perform orthopedic manual therapy. This was completed by open-ended free-text questioning. After completion of Round 1, the data were downloaded by the primary investigator and presented to the workgroup for analysis. First, workgroup members analyzed data entries and developed themes by literal thematic coding methods (coding based on related words or phrases) [1]. Qualitative analysis was then performed to place the remaining data within these categories. Data entries that did not fit into previously created categories initiated a new category being developed. Following individual analysis, the group collaborated and with 100% agreement between the 4 workgroup members were able to move forward into the final workgroup categorization. Following the completion of coding, the workgroup developed recommended statements representing the content within each collective theme. These statements were used to develop Round 2 of the Delphi.

The purpose of Round 2 was to allow participants to rate themes by the level of importance to include or omit from manual therapy educational models. Invitations to participate in Round 2 were distributed via email to those who completed Round 1. Round 2 utilized a 4-point Likert scale to assess agreement with recommendations (Strongly Agree, Agree, Disagree, Strongly Disagree)). The expert panel had 30 days to complete Round 2, with a reminder email at 14 days to promote participation.

After completion of Round 2, the workgroup utilized descriptive statistics to create stacked bar charts to represent all responses. These graphical depictions of Round 2 response along with the same Round 2 questions were re-issued to the participants as Round 3. The purpose of Round 3 was to allow participants to identify themes that they consider to be most important to include or omit from manual therapy educational models while considering the opinions of the other participants.

**Reference**

1. Williams M, Moser T. The art of coding and thematic exploration in qualitative research. Int Manag Rev 2019;15:45-55.
